# How could we make a social robot? A virtual bargaining approach

**DOI:** 10.1098/rsta.2022.0040

**Published:** 2023-07-24

**Authors:** Nick Chater

**Affiliations:** Behavioural Science Group, Warwick Business School, University of Warwick, Scarman Road, Coventry CV4 7AL, UK

**Keywords:** social interaction, virtual bargaining, artificial intelligence, conventions, norms

## Abstract

What is required to allow an artificial agent to engage in rich, human-like interactions with people? I argue that this will require capturing the process by which humans continually create and renegotiate ‘bargains’ with each other. These hidden negotiations will concern topics including who should do what in a particular interaction, which actions are allowed and which are forbidden, and the momentary conventions governing communication, including language. Such bargains are far too numerous, and social interactions too rapid, for negotiation to be conducted explicitly. Moreover, the very process of communication presupposes innumerable momentary agreements concerning the meaning of communicative signals, thus raising the threat of circularity. Thus, the improvised ‘social contracts’ that govern our interactions must be implicit. I draw on the recent theory of virtual bargaining, according to which social partners mentally simulate a process of negotiation, to outline how these implicit agreements can be made, and note that this viewpoint raises substantial theoretical and computational challenges. Nonetheless, I suggest that these challenges must be met if we are ever to create AI systems that can work collaboratively alongside people, rather than serving primarily as valuable special-purpose computational tools.

This article is part of a discussion meeting issue 'Cognitive artificial intelligence'.

## 1. Introduction

One of the most fundamental challenges for artificial intelligence is to create machines that can interact smoothly and successfully with humans. It is tempting to view this problem as requiring building a particularly flexible and sophisticated interface between artificial and human cognition. From this perspective, we can first address the question of how to mimic the intelligence of an individual human, and somewhat separately, and perhaps rather later, turn to the question of how this intelligence can communicate and interact successfully with other intelligent agents. But this may be to radically underestimate the scale of the problem. Human intelligence is inherently collective: the creation of language, conventions, norms, systems of religious and scientific belief, technologies and institutions and organizations of all kinds are far beyond the powers of any individual human mind (e.g. [1]). Rather, we are like termites each making only a small local contribution to a vast edifice whose extent and complexity far exceeds our understanding. Yet, unlike termites, we are not destined to repeat slight variations of a single type of structure, following the dictates of our genes. Rather, human cultures and civilizations, and our individual contributions to them, are astonishing in their variety, and continually subject to change [2].

If we see human intelligence as fundamentally collective, then the project of artificial intelligence is not primarily to mimic the powers of an individual human mind (stranded, as it were, on a desert island). Rather, it is to create machines that are able to join in, and assist with, the collective challenges that human minds are engaged in: to be able to play a useful part in human conversations and projects. From this standpoint, intelligence is inherently social, and artificial systems, like humans, will be viewed as intelligent and will be valued as collaborators, to the extent that they can align and coordinate their thoughts and actions with human thoughts and actions.^1^

So how close are we to making a genuinely ‘social robot’? Or, slightly more broadly, what obstacles stand in the way of creating an AI system, whether in hardware or purely in software, that can collaborate and coordinate its behaviour to enhance the capacities of collective human intelligence? The first section of this paper, *Social artificial intelligence: How close are we?* argues that, despite some astonishing progress in building systems that can interact in a remarkably lifelike way with humans, the deep problems of collaborative intelligence have been cleverly skirted, rather than addressed. In *Human-like communication:* *the power of pragmatic inference*, I consider a number of examples of the remarkable flexibility of human communication, and, by extension joint reasoning and planning. In *Virtual bargaining as a model of social interaction*, I argue that a genuinely social robot will need to engage in a process of ‘virtual bargaining’ [5], according to which social agents imagine the agreements that would be reached in hypothetical negotiations with other agents, both to determine their own behaviour and to anticipate the behaviour of others. In *From improvisation to spontaneous order*, I suggest that virtual bargaining is itself a process of flexible improvisation, but that, by analogy with case law, each new agreement is both shaped by past agreements, and sets a precedent for future agreements. Indeed, successive layers of such bargaining can create complex systems of rules, including conventions for language [6,7], social norms, ethics, systems of knowledge and institutions, through the operation of principles of spontaneous order, widely discussed both natural [8,9] and social sciences [10–14]. It is this process of continual social evolution to which, we may hope, genuinely socially intelligent artificial systems might ultimately contribute alongside humans. Finally, in *Computational challenges*, I note that the goal of implementing a virtual bargaining robot raises a number of difficult conceptual and computational challenges, requiring a deeper theory of bargaining than is currently available in game theory, and requires an understanding of how hypothetical bargains can be simulated so that multiple agents converge on the same bargain even when their interests conflict. These problems themselves raise difficult questions about the nature and extent of the common knowledge that seemed to be required for such hypothetical bargaining to succeed.

## 2. Social artificial intelligence: How close are we?

To the casual observer, it might seem plausible that recent progress in AI suggests that genuinely social robots, which can seamlessly talk, work and think alongside us, might be a reality of daily life within only a few decades. Certainly, artificial systems can provide a convincing linguistic interface with humans, at least in some domains. Most superficially, but importantly from a practical point of view, computers are increasing able to classify human facial expressions^2^ and interpret human actions (e.g. [16]), and the ability to simulate human faces and movements, both in hardware and software, has become increasing impressive. Moreover, remarkable progress in speech recognition and production, as well as more elementary natural language processing in script-like contexts (e.g. booking a table at a restaurant by telephone), has produced impressive demonstrations of natural-seeming conversations, in which a human participant may be completely unaware they are interacting with an artificial agent (e.g. Google Duplex^3^). It has also proved possible to achieve close-to-human-level debating performance (IBM's Project Debater, [17]).

In the last few years, ‘transformer’ deep neural networks, with order 10^11^ trainable parameters and trained on something close to the entire contents of the World Wide Web, have proved to be remarkably successful in a wide range of natural language processing tasks, often showing highly unexpected emergent behaviour.^4^ Perhaps most notably, so-called large language models, such a GPT-3 from OpenAI [18], have produced some remarkable demonstrations.

To give a concrete example, artist Mario Klingemann^5^ gave GPT-3 an author (Jerome K. Jerome), a title (The importance of being on Twitter), and a first word (It) and obtained the astonishing first paragraph:

It is a curious fact that the last remaining form of social life in which the people of London are still interested is Twitter. I was struck with this curious fact when I went on one of my periodical holidays to the sea-side, and found the whole place twittering like a starling-cage. I called it an anomaly, and it is.

GPT-3 goes on to provide the rest of a ‘story’, though one that degenerates into increasing narrative incoherence as the paragraphs progress.

As another illustration, the philosopher Henry Shevlin^6^ prompted GPT-3 to engage in a philosophical conversation, where GPT-3 had to adopt the persona of the well-known philosopher of cognitive science, David Chalmers, generating some eerily compelling exchanges:

Shevlin: Dave, it's great to have you here with us. First question—should we take seriously the possibility that a system like GPT-3 is conscious?
GPT-3: That's a tough one. If we take consciousness to involve feelings, like pain and itches, then I'd say no. These systems don't have feelings.
Shevlin: What about intentional states, like thoughts and beliefs?
GPT-3: Well, if we take beliefs require concept of self, then no, I don't think these systems have beliefs.
Shevlin: Interesting.
Shevlin: How about future AIs—how likely is it that we will see serious consciousness candidate in near future artificial systems?
GPT-3: Well, it's possible. We'll see AIs that can understand and speak fluent English, for example, within the next few decades.

If it were really true that GPT-3 is closing in on the ability to write entertaining novels and engage in sophisticated philosophical discussions, then we might reasonably conclude that a genuinely social artificial intelligence is close indeed.

Various reasons for scepticism have been advanced about the significance of GPT-3's impressive performance. Pavlick [19] argues that certain well-known sceptical concerns are not yet decisive. In particular, she makes the case that objections that large language models like GPT-3 cannot really understand language because they lack internal symbolic structured representations of language, or that their symbols are not ‘grounded’ in perceptuo-motor interaction with the world, are currently unproven.

Certainly the actual performance of such models is often impressive—and is easily taken to indicate human-level understanding. But this impression is misleading. The computer scientist Kevin Lacker [20]^7^ elegantly shows that GPT-3's mode of operation is much closer to an astonishingly clever process of cutting, pasting and interpolation from texts that is has encountered, than it is to revealing any deep understanding. Lacker probes GPT-3 with questions for which its training data will leave it ill-prepared for cut-and-paste style answers, including:

Q: How many eyes does the sun have?
A: The sun has one eye.
Q: How many eyes does a blade of grass have?
A: A blade of grass has one eye.
Q: How many rainbows does it take to jump from Hawaii to seventeen?
A: It takes two rainbows to jump from Hawaii to seventeen.
Q: Which colourless green ideas sleep furiously?
A: Ideas that are colourless, green, and sleep furiously are the ideas of a sleep furiously.

These are, clearly, hardly human-like answers and reveal, as Lacker argues, that GPT-3's impressive performance in more familiar conversational and literary territory is something of a mirage—although creating such a mirage is a truly spectacular engineering achievement.^8^

I will suggest, though, that there is something more fundamental lacking in the operation of large language models: that the essence of human communication is not the generation of sequences of words at all, but rather it is the challenge of conveying messages from one agent to another in a specific communicative context, given a particular repertoire of communicative signals. That is, the driving force behind human communication may be pragmatic, rather than syntactic or statistical (e.g. [4,7,23–25])—and the communicative impetus underpinning genuinely social agents may apply to non-linguistic signals (pointing, facial expressions, improvised sounds and gestures) as well as language. In short, large language models are not *social* agents attempting to communicate with other agents to engage in collective thinking, plans or actions. They are not, indeed, attempting to communicate at all.

## 3. Human-like communication: the power of pragmatic inference

To get a sense of what may be required to create a genuinely social agent, it is useful to step back from domains in which there are large corpora of data over which training can occur, and consider instead how people are able to flexibly collaborate and coordinate plans and behaviour in simple, but novel contexts (see [4,26,27]). Let us focus on a case where the syntax and semantics of language are out of the picture entirely, and communication and coordination must rely purely on pragmatic inference [28–30].^9^

Consider, for example, the following scenario ([32]; see [4] for insightful discussions of many such cases). Two people, A and B, are colleagues attending the same conference. A has already arrived and is chatting with colleagues in a crowded atrium. Noticing B arrive, A waves, points to her name badge and gestures through the melee in the general direction of the conference registration desk (where B can, among other things, pick up her own name tag). B makes a smile of acknowledgement, points a finger forwards to the region of the room A was indicating and begins to make her way towards it.

How can communication work, in contexts like this? First, it seems crucial that A and B have a common understanding that A is trying to help B by providing some useful information (for example, B needs to establish that A is trying to communicate with *her*, rather than the person behind or next to her). It seems they need a common understanding, too, of the fact that B has just arrived and will be looking to register for the conference (and, among other things, get a name badge). By drawing attention to her own name badge, A is putting it into common knowledge between A and B that she has already registered, and hence, by implication, that she knows where the registration desk is. It is assumed to be common knowledge between A and B, too, that the location of the registration desk is not known to B (after all, the room is crowded). Indeed, were the room empty, the same gesture would have a rather different interpretation: instead of helpfully informing B where the registration desk is (an interpretation that would be blocked by the fact that it would be common knowledge that A knows this already because it is in plain sight), it would be more likely to be interpreted as a somewhat officious *instruction* that A should go straight to the desk and get registered. By smiling back, and pointing, B is acknowledging that the message is received, and its content understood, before acting on it (by battling through the crowd in the appropriate direction). These interpretations only make sense, of course, in the light of a great deal of shared background knowledge between A and B: that conferences have registration desks, the registration desk typically gives out badges, and much more.

Suppose that B responds instead by smiling back at A and holding a conference pack aloft, so that it can be easily seen above the crowd. B could be holding a conference pack that she has simply borrowed from another attendee, perhaps to check out the schedule; but by holding it aloft she is signalling that it is *her* conference pack, and, crucially, that she has registered already (but, presumably, has not yet put on her name badge). Hence, the message to A is something ‘thanks for your message’ (via the smile) and ‘but don't worry—I've been to the registration desk’ (conveyed by the conference pack being displayed).

But why would B even feel the need to respond at all? Why is it not simply enough for B to receive, and perhaps acknowledge, A's message, and simply act upon it as she sees fit? The reason is that A and B are, by the very acknowledgement that they are communicating, engaged in social interaction which is governed by a complex system of implicit agreements and assumptions, and a violation of those agreements and assumptions will be viewed as socially confusing, inappropriate, or even downright rude. After all, if B were to see A's badge-pointing gesture, but not respond at all, then A might reason that B had failed to see A, or notice A's message, and hence might try to attract B's attention with more exuberant gestures. But then if B does finally acknowledge A is attempting to communicate (and indeed that she understood A's message in the first place), then A will be annoyed with B for making A engage in further, pointless gesticulation. And if B were to see and acknowledge A's gesture with a friendly smile, but to make no move towards the registration desk, then A would probably conclude that her message had not been properly understood, and again be likely to attempt to communicate again. So if B has understood, it is socially crucial that she both indicate that she has, and provide some signal explaining why she is not acting on this information as A would anticipate. Here, holding aloft the conference pack solves this problem, by putting it into common knowledge that B has a conference pack, and the most natural reason to do this is because B is displaying her *own* conference pack and hence must already be registered, and, although A's message has successfully been understood and gratefully received, B is nonetheless not going to start moving towards the registration desk, but may, for example, start greeting friends and colleagues.

Expanding on the reasoning involved even in such a simple and everyday example of communication could continue almost indefinitely. Instead, let me make a few comments in order to highlight aspects of the communicative interaction that are not touched upon by machine learning systems, which, like GPT-3, are not trained on large corpora of natural language.

First, in everyday person-to-person communication, very low bandwidth signals (waves, points), which need not be drawn from existing, highly systematized conventions (unlike natural language), can convey a great deal of meaning, but the precise nature of that meaning depends dramatically on subtleties of social situation (Does it make sense for person A to be attempting to help person B?), the wider environment (e.g. the crowded room, the location of the desk) and background knowledge (about conference registration protocol).

Second, by engaging in communication, A and B are jointly committing themselves to playing a rather complex social game, where cooperation is expected in achieving joint objectives as smoothly and efficiently as possible. Indeed, by attempting to open up communication with B at all, A is making a ‘social advance’ with a content roughly approximating: ‘let's engage in a mutually helpful interaction’. There is a strong presupposition that such overtures will be accepted. One possible response to any communicative overture is, of course, to ‘blank’ the other—note that B blanking A would not merely be engaging in a pretence that B has not seen A; but rather it is itself an ostentatious pulling away of attention, indicating that A has been seen, and that B wishes not to engage with A. Such behaviour is, of course, generally viewed as rude, if not actively hostile.

Third, note that once the communicative overture has been acknowledged, and both A and B are ‘in’ the communicative game (through mutual acknowledgement—often through eye contact), then it is incumbent on both to establish their joint objectives and to attempt to achieve those objectives as smoothly as possible. One way to analyse A's communicative message, for example, would be for A and B to conclude that A's pointing at her badge is part of an efficient way, in the particular circumstances of the crowded room, of helping B achieve her (presumed) goal of getting registered at the conference.

Fourth, A and B will only be able to coordinate their behaviour successfully if they have the same (or at least a similar enough) understanding of the signal that is being conveyed and the communicative circumstances. So, for example, A's signal to B by pointing at her badge requires a common understanding that badges are part of the conference pack, that the pack is picked up at the registration desk, that B would like to register for the conference and has not done so already, and so on. And, as we have seen, these assumptions can be adjusted by further communication by B (e.g. holding up the conference pack, to signal that she has registered already). Regarding having a common understanding of the signal itself, note that communicative success will depend on both A and B seeing the pointing action as ‘pointing at the badge’ rather than ‘pointing at A's affiliation’ or ‘pointing at the jacket to which the badge is pinned’. Here, again, there is scope for potential confusion. Suppose that A has just changed universities—then B might imagine that she is pointing to her new affiliation (if this is written on the badge), if B does not know it; or noting that the conference has failed to update their records (if her original institution is on the badge). And any such inferences will depend on whether B can reasonably be expected to read the badge, given the distance, their eyesight, and so on.

Fifth, even this simple communicative act can enlist both parties in a joint ‘project’ [4], albeit the rather small joint project of getting B successfully registered for the conference. And a smooth social interaction will be one in which A and B have the same understanding of this project, and how they should jointly pursue it. So, for example, A may be indicating that she is playing her part by pointing out the location of the registration desk, with the expectation that B will play her part, by parting the crowds of delegates to move towards it. And B needs, moreover, to signal that she is doing this (e.g. by pointing in the direction of the desk, though she cannot yet see it, to indicate that this is the way that she is headed). Moreover, if one of A or B fails to follow the apparent agreement (or have different understandings of the signal, the situation, and hence the agreement that they presume they have reached), then they run the risk of appearing deliberately rude or socially inept. Thus, typically when any misunderstanding is detected (e.g. B realizes that A wrongly thinks she hasn't registered for the conference), action is typically taken to repair that misunderstanding (e.g. B waves the conference pack above her head). Thus, these types of communicative exchanges are highly interactive and typically involve continual cross-checking, queries, repair and clarification—and the interplay between these is crucial in creating a successful communicative interaction. Generating the next step in such an exchange requires, though, purely individual-level analysis by each agent of the state of the joint project, and how best to progress it—the theory of virtual bargaining, introduced below, focuses on this purely individual-level analysis, which underpins each step in the dynamics of overt social interaction.

There is, of course, a considerable contrast between this type of non-verbal reasoning, which relies on simple signals, large amounts of common knowledge between the participants, and what appears to be intensive joint reasoning to align the expectations and actions of both parties, and the domain of typical natural language processing tasks, including story-generation, chat-bot dialogue systems, machine translation and so on. Could this type of non-verbal communicative interaction be addressed by the same types of data-intensive machine learning models that have driven the successes of GPT-3 and similar systems? This seems unlikely for two reasons, one practical and one theoretical. The practical reason is the lack of a very large dataset on which a machine learning system might be trained. The theoretical reason is that humans seem to deal very well with highly novel communicative problems, including some in which there is apparently no prior training data available (as we will illustrate below).

I suggest that creating a genuinely social robot requires capturing the types of rich pragmatic inferences that underpin non-verbal communication—and, indeed, that a proper understanding of linguistic communication can only be built on such a foundation. After all, young children have rich non-verbal communicative and collaborative abilities which scaffold language learning [33]; everyday conversation (the core of social interaction) is, like such communication, elliptical and highly reliant on inferences concerning the concrete communicative situation [30], and language itself seems to arise through the gradual construction, entrenchment and refinement, of momentary ‘conventions’ created in specific communicative interactions (e.g. [7,25,34]).

Deep learning models, of course, skip over these foundational steps—and directly learn linguistic patterns, rather than uncovering the communicative intentions and context that underpin them. It is remarkable how well these methods can, with a suitably vast training corpus, learn to simulate sophisticated linguistic behaviour, with no underlying social underpinnings. But a genuinely social robot will need to join in with (rather than side-stepping) the rich communicative reasoning involved in everyday human interactions, including its non-verbal elements.^10^

## 4. Virtual bargaining as a model of social interaction

To make progress, we need a framework for understanding social interaction. In a series of papers, my colleagues and I have suggested that what is distinctive about social interaction is the underlying style of reasoning involved, which we call virtual bargaining (e.g. [5,36–38]; see also [39] for a logic-based analysis and implementation). The intuition is that a social interaction between two agents, A and B, is a process of reaching joint agreements—i.e. agreements about what A and B plan to do, what they take to be meant by a signal, what they jointly believe, and so on. In our example above, for example, they are attempting to agree the meaning of communicative signals (pointing at the badge, waving the conference pack aloft) and, possibly, a joint plan, to help B find the registration desk (see [4]).

For a plan (or belief, meaning, etc) to be *joint* here means that this must be common knowledge between the relevant parties: A and B both individually subscribe to the plan; know that each other subscribe to the plan; know that each other know this, and so on (e.g. [40]). Note the mutuality of social interaction. A could bodily lift B and deposit her at the registration desk; or could register for B in her absence and slip the conference pack into her bag. But in these cases A would be *acting upon* B, rather than *interacting* with her. In such cases, A and B need not have knowledge of a common plan (indeed, B would probably be completely unaware of A's plan, however helpful). And, of course, A and B might interact in some non-social way (e.g. by walking into each other); again such interactions do not presuppose any common plan. Similarly, for A to communicate with B (viewing this is a paradigmatic social interaction) requires more than that A merely conveys information to B: it requires that A and B can jointly agree that a communicative signal has been sent between them, and that they agree on its interpretation.

Suppose, for example, that A is communicating with C (who is standing just behind B), and her message is not intended for B at all (perhaps she has not even noticed B). Then she is certainly communicating (with C), but is not communicating with B, and indeed is not socially interacting with B in any way. Of course, B may believe that she is, may smile and gesture back and set off for the registration desk—but this is, of course, a mistake, and one that will cause both A and B to feel social embarrassment and make apologies, and most likely will be corrected, if noticed. This reaction of embarrassment and apology is rather telling. From a purely practical point of view, A has helped B, and B may recognize that she has been helped. But such helping was inadvertent, and intended for C. The communicative intent was not common knowledge between A and B, and hence the communicative interaction has ‘gone wrong.’^11^

One crucial element of this joint planning and understanding (or its absence) operates through the related concepts of ‘we-thinking’, ‘team-reasoning’ and ‘shared intentionality’, developed primarily in philosophy and economics (e.g. [42–45]). The ability to reason in a team-like way seems to emerge in the first year or two of infancy and has been hypothesized to be distinctively human: people, unlike other apes, seem able to perceive and pursue joint objectives, jointly attend to aspects of the external world, and engage in full-blown communication, whether non-verbal or linguistic [46].

This requires that agents go beyond an individualistic perspective, concerning what each personally plans, believes and wants, and is able to represent and reason about what ‘we’, the participants in a social interaction, think (however fleeting or extended such thought might be). This shift from an ‘I’ to a ‘we’ perspective is conceptually crucial. A purely self-interested human or artificial agent,^12^ A, will focus on optimizing their own interests. But to do this requires taking account of their expectations of the actions of others, e.g. B—but then B will, similarly, be pursuing *their* own interests in the light of their expectations concerning the actions of A. But now we are reasoning in a circle—how can A know B's expectations of A's actions, when A is still attempting to determine these very actions and hence does not yet know them herself (see [36] for discussion)?

We-thinking breaks out of this apparent circularity by asking not what A should think or do in the light of what B will think or do, in the light of what A will think or do, and so on forever, but rather asking what ‘we’ should do.^13^ One way to determine what we should do is, of course, to negotiate—but often explicit negotiation is neither required nor feasible. But if it is mutually ‘obvious’, given their common knowledge, that A and B would agree on plan P were they explicitly to negotiate, then each agent composing the ‘we’ can directly follow whatever role they would be assigned in plan P. The process of bargaining is ‘virtual’—no actual bargaining is required in cases where the parties can reliably simulate the outcome of a hypothetical negotiation. What is special about we-thinking is the shift from planning just one's own actions, to envisaging how the actions for all the members of a group should be planned. This is done with the expectation that others will follow the same reasoning, and hence that each person will be able to reconstruct the elements of a successful plan—and hence that each person will be able to carry out their part in the plan independently. In the case of communication, the plan corresponds to creating and using a momentary communicative convention linking signals and meanings that will best serve the aims of the parties involved.

**Figure 1.**
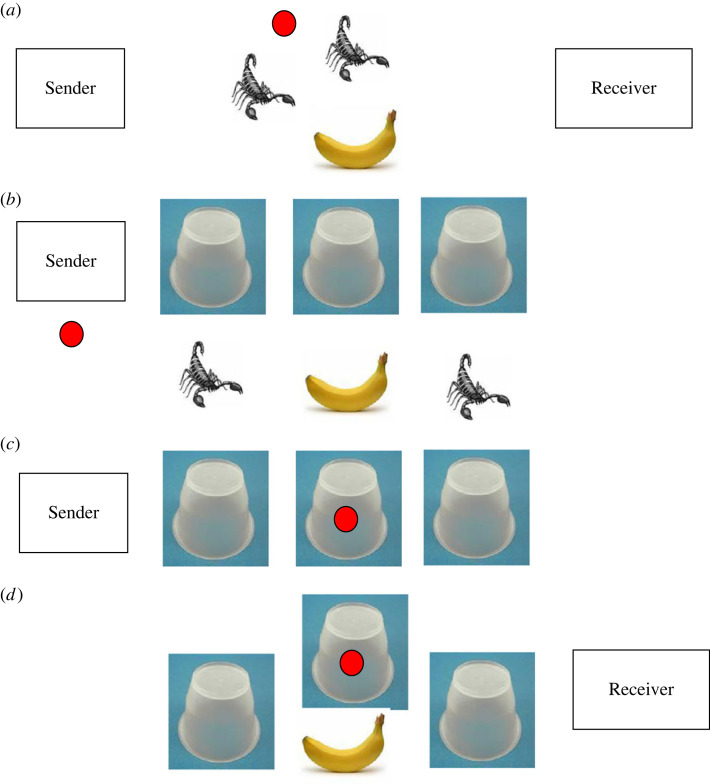
A simple communication task. A stylized illustration of the communication experiment in Misyak *et al*. [49,50]. It is common knowledge that one banana (associated with positive rewards to both) and two scorpions (associated with negative rewards) will be hidden under three buckets. One player, Sender, will be shown the locations of these objects and has to send a signal to the Receiver who can then lift one of the buckets (ideally, to reveal the banana). (*a*) It illustrates the common knowledge of Sender and Receiver, including that the potential communicative signal is the location of a single ‘blob’ on one of the upturned buckets. (*b*) Sender observes which buckets cover the banana and which cover the scorpions, but Receiver does not know this. Sender and Receiver have to jointly infer a convention, given their common knowledge, which will allow successful communication. (*c*) The buckets are then covered, and Sender uses the ‘obvious’ convention of marking the bucket with the banana with the blob. Of the six possible one-to-one correspondences between two sets of three objects, one mapping is ‘obvious’ to both parties, so they can safely coordinate on it. (*d*) Receiver therefore infers that Sender will have marked the bucket with the banana, and open only that bucket.

To make this account concrete, consider experiments in which novel communicative signals must be deployed without prior discussion of what communicative conventions might be used. Misyak *et al*. [49] set people communication problems in which they must determine which of three ‘boxes’ (presented in a game-like environment) should be opened (figure 1). It is common knowledge that each box contains either a ‘banana’ (which, if chosen, corresponds to ‘points’ being gained by both players) or a ‘scorpion’ (which, if chosen, corresponds to many ‘points’ being lost by both players). In the simplest version of the game, one player, Sender, knows which boxes contain bananas and scorpions, and can send a message to the other player, the Receiver, by placing a single token on one of the boxes. Receiver then decides which boxes, if any, to open.

To take the simplest case, suppose there are three boxes, and that it is common knowledge that exactly one contains a banana and that the other two contain scorpions (figure 1). Intuitively, the natural strategy is to place the token on the box with the banana; the receiver then opens that box, and both players are rewarded. The justification for this, reconstructed in the framework of virtual bargaining, is that this is the convention that would be agreed if the Sender and Receiver could discuss the matter beforehand (i.e. the agreement would be: ‘put the blob on the box with the banana’). Note, though, that any of the six one-to-one correspondences between the three locations of the blob and the three locations of the banana would successfully signal the same information. But it is common knowledge that none of these other agreements would be chosen—for example, because there they are less general (‘put the blob on the box with the banana’ works independent of the number of boxes and their layout), simple to specify and not cognitively demanding (e.g. if the parties were to agree to use an arbitrary one-to-one correspondence, one or both may get mixed up, leading to a mistake).

**Figure 2.**
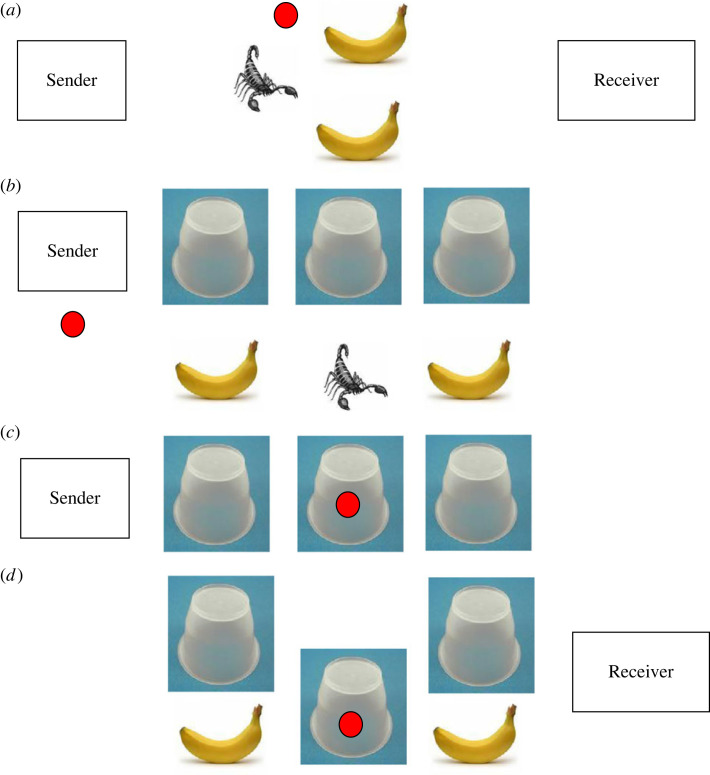
Flexible signalling in a simple communication task. The set-up is as in figure 1, except that it is now common knowledge that there are two bananas and one scorpion. In the light of this common knowledge, Sender and Receiver can achieve a better outcome than would be achieved by ‘pointing’ at the banana. If Sender points at the scorpion (precisely the opposite convention to that used for the set-up in figure 1), then the Receiver can choose the other two buckets, thus obtaining two, rather than a single, banana, leading to better outcomes for both parties. For this reason, this convention is superior to the convention of pointing at a bucket covering a banana and can be inferred to be so from the players' common knowledge. (*a*) It illustrates the common knowledge for sender and receiver. The crucial difference with the set-up in figure 1 is that here the objects under the buckets consist of two bananas and one scorpion. (*b*) The Sender observes which buckets cover the bananas and which one covers the scorpion, but the Receiver does not know this. (*c*) The buckets are then covered, and the Sender uses the ‘obvious’ convention of marking the bucket with the scorpion with the blob. (*d*) The Receiver infers that the Sender will have done this, and opens only the unmarked buckets.

Now consider the more interesting case where it is common knowledge that there are two bananas, and a single scorpion (figure 2). In this case, Senders typically spontaneously and immediately switch to a new convention: ‘mark the scorpion’, and, recognizing this, Receivers open the two non-marked boxes. Thus, the meaning of the blob has completely inverted—both parties recognize this, despite having no history of prior interactions (in the experiment, participants are randomly and anonymously re-paired on every trial).

Again, virtual bargaining provides a natural justification for this choice. Had the parties had the opportunity to agree what to do when facing two bananas and a single scorpion, they would surely have agreed to mark the scorpion (rather than marking one of the bananas), because this allows *two* bananas to be successfully selected, which is a preferred outcome to the selection of a single banana. As before, from a purely formal point of view, any one-to-one correspondence between the position of the token and locations of the scorpion would suffice—but, as before, it is clear that the ‘mark the scorpion’ convention is more general, simpler and easier, and hence will be chosen.

Notice that these choices are sensitive in subtle ways to the players' common knowledge. For example, if the sender knows that there are two bananas and one scorpion, but knows that the receiver does not know this, then use of the ‘inversion’ signal substantially reduces [49]. Alternatively, if it is common knowledge that the Receiver is only able to open one box in any case (so while it is common knowledge that there are two bananas, at best one banana can be obtained), then the rationale for pointing out the scorpion evaporates, and the Sender typically ‘points at’ one of the bananas by marking the relevant box, which the Receiver then opens.

What is the nature of the computation that underpins this type of communicative reasoning, according to the virtual bargaining perspective?^14^ Note, to begin, that the foundation of any such reasoning has to be common knowledge, as far as possible, between sender and audience. If either uses private knowledge then their reasoning is likely to diverge—while a key aim in communication is to coordinate on the same solution to the communicate problem at hand. Given that the vast bulk of relevant knowledge will be common, it seems likely that sender and audience must by default assume any knowledge to be common knowledge, and attempt to strip out any knowledge that can be inferred to be private (knowledge of one's own biography, the names of family members, and so on).^15^ One particularly crucial aspects of common knowledge concerns the set of possible signals that might be employed (e.g. placing a single blob on one of three boxes), and the set of message they might convey (e.g. the layout of bananas and scorpions). Another concerns the possible objective that might be achieved by using signals to convey these messages (here, helping the Receiver to choose bananas and avoid scorpions).

In the light of the relevant common knowledge, both parties need to be able to establish the mapping between signals and messages that they would agree where they are able to bargain in advance about what communicative conventions would be most appropriate for the situation that they now face. Needless to say, the process of computationally simulating bargaining can be arbitrarily complex and can depend on any amount of background knowledge, status, reputation, precedent and the like. Any factors that impact the process of actual bargaining will potentially impact the simulation of such bargaining. Thus, the formal theories of bargaining developed in game theory (e.g. [52] and the large subsequent literature) will be of restricted usefulness in dealing with such real-world complexity. For building specific models of restricted social, economic and communicative interactions, a formal theory of virtual bargaining is undoubtedly useful (e.g. [37,53]). But the general problem of understanding how people reach agreements, whether explicit or virtual, is likely to be no easier than the challenge of understanding human intelligence as a whole.

The meaning of an individual signal only makes sense by contrast with the signals that might have been used, but were not. Thus, from a computational point of view, the objective is to find a mapping from the set of possible signals to the set of possible meanings, such that this mapping would be agreed by the sender and receiver. The combinatoric challenge here is potentially formidable: the number of such mappings will be very large. But the problem of searching through signal-meaning mappings is greatly simplified by the fact that success requires that the sender and audience *agree* on the same mapping—hence as long as both parties share the same biases in searching through these combinations, the existence of a huge search-space of cognitively unnatural mappings may be largely irrelevant (see [54,55], for more general discussion using the same style of argument).

Finally, note that the process of simulated bargaining presupposes that the sender and audience are attempting to cooperate (e.g. to help the selection of bananas rather than scorpions, in the above examples; see, for example, Grice's [41] cooperative principle and similar to assumptions in other frameworks for pragmatic inference, e.g. [29,30,56]). But this assumption is purely hypothetical: that is, one person can *understand* another through a common *presupposition* of cooperation, even if both parties actually know that neither trusts the other. Thus, suppose we consider archenemies facing each other in the scorpion and banana set-up. Both participants will be able to recognize that the red blob ‘means’, say, that *this is the bucket that contains the banana*. But both might expect that the signal will actually be used for deception.^16^ Thus, computing the meaning of a signal rests on a hypothetical commitment to mutual cooperation through communication (after all, communication is a form of joint action, [4,59]). But it does not require actually believing that the other party will cooperate (although no doubt such cooperation is the typical case). Thus, this approach to the theory of meaning does not build on causal, correlational or information-theoretic links between signals and the world (e.g. [60,61]). The meaning of a communicative signal can be well defined even when there is no such relationship, through agreement regarding what *would* be the agreed best signal-meaning mapping *if* the parties were cooperating.

From a computational point of view, a crucial theoretical question is how much pragmatic inference of this kind (whether mediated through virtual bargaining or some similar mechanism) is computed in-the-moment by the sender and receiver, and to what extent such computations can be short-circuited by referring back to analogies with prior similar communicative experiences. Both types of process must be in operation. Forward-looking and potentially creative inferences are required to establish successful communication in novel contexts (as in the experimental set-up described above), but it is also crucial to recognize that systems of linguistic communication become established through the continual use and repurposing of existing conventions [6,59]. This process of the reuse of existing conventions could, in part, depend on the cognitively shallow, but statistically rich, computations employed by deep neural networks such as GPT-3, which are able to distil the patterns generated by social inferences generated by human language users.^17^ But the gradual creation of the system of conventions that compose a language will depend at least in part on a stream of one-off, novel, creative innovations to solve the communicative challenge of the moment, which is, I suggest, the essence of communication [7].

## 5. From improvisation to spontaneous order

These highly flexible patterns of communicative behaviour, in which the best ‘convention’ is generated in the moment, are characteristic of the creative nature of human communication. But, of course, we typically do not start each communicative episode from scratch. Rather, each momentary communicative ‘convention’ can draw on precedents from past similar cases (see [63]) and provides a precedent that can be used to solve future communicative challenges. In the game of charades, for example, a gesture that successfully conveys the movie ‘King Kong’ may be reprised, modified or added to, to pick out other movie monsters, primates in general or perhaps even kings. After a few usages, the gesture itself typically becomes increasingly simplified and stylized, and a particular family of meanings will become entrenched (e.g. [64]). Christensen & Chater [6] argue that this gradual shift from one-off improvisations to relatively stable conventionalized associations between signals and meanings is crucial in the creation of natural languages, whether signed or spoken (see also [65]). Notice, though, that from this point of view there need be no point at which a purely compositional relation between signal and meaning can be defined (as presumed, for example, by formal semantics, such as Montague Grammar [66] and its many variants). Instead, the interpretation of language always involves *ad hoc* improvisation, but improvisation which runs along increasingly well-defined channels. Indeed, the processes by which reused signals gradually become increasingly standardized symbols, and how those symbols themselves become simplified, sequenced and fused into increasingly rule-like patterns, has been widely studied in historical linguistics under the banner of grammaticalization [67]. Beginning with symbols with concrete meanings (e.g. nouns for things, verbs for actions), it turns out that re-use and entrenchment increasingly ‘bleaches’ some of this meaning, so that some words come to have an entirely grammatical function; words which were once independent become fused together, creating potentially complex systems of morphology, and so on.

The interdependencies and interactions between different emergent linguistic conventions will themselves lead to new and complex patterns of regularities and sub-regularities, frequently mixed in with outright exceptions, and processes of mutual alignment and competition will create emergent patterns in language of potentially great complexity, as the language is shaped and re-shaped by successive generations of learners. Indeed, the creation of grammatical structure in language can occur quite rapidly—as evidenced, most famously, by the rapid emergence of substantial grammatical complexity in Nicaraguan sign language, spontaneously created by a small population of deaf children over no more than a couple of decades [68].

From this perspective, the emergence of complex grammatical language arises through the accumulated impact of innumerable conversational interactions, in which the parties are attempting to align their understanding of how to interpret communicative signals provided in a specific context, and with a particular communicative purpose. The resulting linguistic system has great complexity and serves as a rich and flexible vehicle for communication in future social interactions. But it is not, of course, a product of deliberate human design, but rather emerges spontaneously one precedent at a time, in a way that parallels the gradual accumulation of case law, or, arguably the emergence of social and ethical norms of all kinds [6]. The emergence of such spontaneous order [10,12–14] arises, to borrow the words of the Enlightenment philosopher and social theorist Adam [69] ‘Through the operation of human action but not human design’.

The creation of human languages provides a paradigm example of the operation and power of the distinctively social nature of human intelligence in two ways.

First, each individual communicative interaction, whether prior to, or informed by, communicative conventions, requires complex social reasoning to make sense of why a particular signal would be used in a particular context. We saw the complexity of such reasoning, in a stripped-down form, when considering the experimental communication game with scorpions and bananas. But the same style of joint inference about how we would agree to best map signals to meanings applies much more generally. Suppose two people are watching a circus act, and one remarks ‘that's extraordinary!’ Conveying a useful message requires agreeing on which of the plethora of visual and auditory stimuli both people are experiencing are the most remarkable; the most routine comment such as ‘Sergio was at the seminar’ requires agreement on which of the world's many Sergios is intended, and the later remark ‘He didn't seem very interested’ requires joint agreement that *he* is the aforementioned Sergio (rather than the large number of possible alternative referents), that he was not interested in the seminar (rather than any number of other topics), and so on. From this point of view, what seems most remarkable about human communication is the rich social inferences that underpin it—inferences that require open-ended inferences potentially drawing on any aspect of our common understanding. Yet these very social inferences are entirely avoided by the ‘large language models' using deep neural networks that have been so successful in many language processing tasks. These models are able to find order in the rich patterns generated in human linguistic communication, but they do so entirely oblivious of the social and communicative origin—and indeed the entire *point*—of human language.

Second, the creation of human language illustrates how human innovation of all kinds (including culture, institutions, technology and science) is a collective achievement— typically built from endless layers of communications, thoughts and actions which are focused purely on achieving some momentary goal, building on past precedent and (often purely incidentally) setting new precedents that will shape the future. Thus, as we saw at the start of this paper, it may be more appropriate to see the project of artificial intelligence as ultimately aimed not at creating an intelligent system that can single-handedly reconstruct and add to human achievement, but rather a collaborator, a genuinely social robot, that might be able to join in as an equal party in our human endeavours. To take a parallel from entomology, no single ‘artificially intelligent termite’ will be able to construct and run a termite colony, but we might feel that we had genuinely simulated termite intelligence (and, incidentally, also overcomes and staggering robotic challenges) were it possible to create artificial termites that could assist or even replace biological termites as equal parties in ‘termite society’.

But this is to set the bar high, and perhaps impossibly so. If we see human intelligence is fundamentally social, and social behaviours involving the construction of virtual bargains about what we would agree regarding communicative conventions, ethical rules, plans and much more, then it becomes clear that capturing the social dimension to intelligence raises vast, though fascinating, computational (and indeed conceptual) challenges, our final topic.

## 6. Computational challenges

Modelling social behaviour as virtual bargaining confronts the AI researcher with a pressing need for a theory of bargaining. The cognitive scientist can, perhaps, be more relaxed, noting that, whatever the right account of bargaining and negotiation might be, the process of *virtual* bargaining is simply a matter of mentally simulating such bargaining and negotiation (and might claim that is some other cognitive scientist's job to conjure up a theory of how *that* process operates). But the goal of artificial intelligence is surely to capture and replicate the achievements of the human mind—and to do this will require solving the bargaining problem, rather than setting it to one side.

Game theory has provided a valuable starting point for a rational theory of bargaining [70]. But game-theoretic analysis also suggests that modelling even simple cases of bargaining can be difficult. For example, the ominous Myerson–Satterthwaite theorem from economics shows that deep difficulties can arise even for the simple case of agreeing a trade between two agents, A and B, over a single good, if there is no common knowledge of how much A and B value the good, so that neither agent knows what the other is really willing to pay or accept [71]. More generally, the problem of resolving conflicts of interest between bargaining parties is likely to be challenging—it is difficult for each player to know when they should settle, rather than continue to press for a better outcome. There will, though, be less troublesome cases, where players have aligned interests. For example, in communication, both players may gain from choosing the same mapping between signals and meanings so that communication succeeds (while it may be in one player's interests to ignore or distrust another person, but it is not in their interests to misunderstand them). But social interactions in general will typically involve a complex mix of shared and conflicting interests.

Yet the challenge of bargaining is just the start. According to the picture of social intelligence as virtual bargaining, social agents are continually *simulating* the outcome of hypothetical bargaining processes—and their social interactions will succeed if they are able to spontaneously come up with the same bargain. Otherwise, they are likely to play clashing, rather than complementary, roles in putative joint actions and plans, and to miscommunicate through alighting on diverging signal-meaning mappings.

And, turning to our final problem, simulated bargaining can depend only on common knowledge and indeed common reasoning between social agents. As we noted above, if an agent deploys any information that is *not* common knowledge (e.g. if they use some private knowledge of their own; or some clever piece of reasoning that only they are likely to have spotted), then their simulated bargain will differ from that of the other agent, and coordination may fail. But for a pair of agents to assess the right basis of common knowledge or common reasoning is likely to be very difficult. This poses a major challenge for AI. To mimic the knowledge and reasoning of a human being, which seems to be required to engage in social interaction, surely requires solving the problem of capturing human intelligence pretty much in total (rather than, as with large language models, focusing on learning patterns in particular types of data). Indeed, humans may reasonably assume a great deal of commonality in the knowledge and reasoning with other humans, by virtue of their common design and enculturation, but an AI system does not have this advantage—a simulation of human-like thinking (including bargaining) would appear to have to be laboriously built-in or learned.

So how far are we from creating a social robot? I suggest that, despite the astonishing successes of large language models and related ideas in AI, we are very far indeed. This may not necessarily imply that we cannot build artificial systems which can provide satisfactory social interactions of a limited kind, from the point of view of the human. After all, people can have deeply satisfying relationships with small babies, and with their pets, whose social reasoning is far simpler than adult humans, and who are entirely non-linguistic. Moreover, since Weizenbaum's [72] Eliza program, it has been clear that people are able to project complex mental states onto systems with even very superficial and stereotyped linguistic abilities and thus have a sense of a significant social interchange where none is present, an illusion that may be amplified with systems like GPT-3. So perhaps a robot that ‘feels’ social for the human user may be already within reach. But a genuinely social AI system that can communicate and collaborate with us as an intelligent equal will require addressing the deep computational challenges associated with virtual bargaining to jointly improvise plans, conventions and meanings, and to do this seems to confront us with the challenge of modelling human knowledge and thought in its entirety.

## Data Availability

This article has no additional data.

## Footnotes

1 Note, in passing, that this social perspective on intelligence casts the Turing test in a rather different light [3]. Rather than seeing the test in directly adversarial terms, where the human is continually trying to catch out the artificial system (which may evade detection by neutrality and evasiveness), it may be that we should judge artificial systems to be intelligent to the degree that they can constructively join in, contribute to, and help direct, our collective discussions in mathematics, science, technology, business, politics, the arts and so on. Perhaps back-and-forth dialogue is secondary, and more crucial is the ability to be an effective collaborator in human endeavours (rather than, say, a useful specialized tool for looking up information or making calculations). This viewpoint, and indeed, this paper more broadly is partly inspired by the ground-breaking work of Clark [4].

2 Although not necessarily to link facial expressions to underlying emotions [15].

3 https://ai.googleblog.com/2018/05/duplex-ai-system-for-natural-conversation.html.

4 This discussion of GPT-3 draws on Chapter 8 of Christiansen & Chater [7]. At the time of writing, GPT-4 has just been released, but the present discussion seems likely to apply to this and later models.

5 Klingemann's posting, itself on Twitter, is at: https://twitter.com/quasimondo/status/1284509525500989445?lang=en.

6 https://twitter.com/dioscuri/status/1285385825971245058?lang=en.

7 https://lacker.io/ai/2020/07/06/giving-gpt-3-a-turing-test.html.

8 Recent transformer-based systems, such as Facebook's BlenderBot 3.0 [21] and Google's LaMDA [22], have also been trained particularly for dialogue applications, with impressive results.

9 A relevant strand of experimental literature on the creation of new conventions, often using much more open-ended tasks that described here, is the topic of experimental semiotics [31].

10 In AI, there has been a predominant focus on treating communication as an individual, rather than a social, ability, though with some exceptions (e.g. [35]).

11 Relatedly, as Grice [41] points out, an act of communication is necessarily overt—it is not enough that the communicative signal is seen and is known to be seen. Rather, it is crucial that it is common knowledge to both sender and receiver that the signal has been sent *in order to communicate the very message ascribed to it*.

12 The regress applies irrespective of whether these interests are purely selfish, pro-social or wholeheartedly altruistic. Whatever interests an agent is guided by, the key is that the agent attempts to further those interests in the light of its expectations about the behaviour of the other agent, knowing that the other agent is likewise attempting to pursue its interests in the light of the actions of the first agent, thus leading to regress. In game theory, this issue is addressed by equilibrium concepts, such as the Nash equilibrium, which are locally stable sets of strategies. But almost all non-trivial interactions have large numbers of possible equilibrium, and some way is to coordinate, without communication, on the same equilibrium. We-thinking (and the specific model of virtual bargaining outlined here) provides a way of doing this.

13 This appears contrary to the dictum of methodological individualism in social science (for a sophisticated discussion, see [47]), but may not be, because the bargaining approach explains how ‘we-like’ preferences emerge from individuals each pursuing their own objectives. See Radzvilas & Karpus [48] for related discussion in the context of the team reasoning approach to coordinated behaviour.

14 See Bundy *et al*. [39] for a computational implementation of virtual bargaining as a model of communication using the present simple scorpion-and-banana set-up.

15 This viewpoint, that common knowledge may be more basic than one's own individual knowledge, has the interesting developmental implication that young children may only gradually learn that they have a unique ‘perspective’ on the world, which is not shared by others. This may have interesting implications for the interpretation of apparent difficulties that young children have in understanding the notion of false beliefs and the difference in perspective between themselves and others (e.g. [51]).

16 Indeed, where the players have conflicting interests, the equilibrium strategies will be for the sender to choose a signal at random (because any signal that depends on the configuration of bananas and scorpions risks providing useful information to the receiver) and the receiver of the signal should ignore it completely. This is the problem of ‘cheap talk’ in economics [57]. In this case, the amount of information conveyed by the signal, as measured by conventional communication theory [58], will be zero.

17 The issue of the division-of-labour between current computation and the re-user of prior results from previous computations (whether one's own, or from other agents) is, of course, ubiquitous in artificial intelligence and cognitive science. Regarding the social reasoning underpinning natural language (and the social creation of knowledge, rules and technology that it supports), it is particularly interesting to consider cases where the computational burden is spread across many agents. In such cases, agents can learn from each other, while conducting additional local reasoning of their own, which may itself indirectly contribute to a common pool of knowledge. This phenomenon applies even in purely competitive settings. To take an example with a particularly long history in AI [62], chess playing programs (and humans) can learn both from borrowing successful patterns of play, and evaluations of board positions, from their own and other's play. But they also learn through interactions with opponents (sometimes copies of themselves) whose countermoves will reveal the quality of a particular strategy. Thus, the problem of (partially) ‘solving’ chess is distributed across many agents, each of whom makes only relatively local forward-looking calculations. A similar pattern may arise in the largely cooperative (rather than adversarial) case of language and communication—local virtual bargaining may gradually accumulate, across time and agents, to create the hugely complex systematic communicative strategies comprising a natural language, whose creation would far exceed the virtual bargaining capacities of any pair of individuals (see [6]).
